# Meteorological factors and risk of hemorrhagic fever with renal syndrome in Guangzhou, southern China, 2006–2015

**DOI:** 10.1371/journal.pntd.0006604

**Published:** 2018-06-27

**Authors:** Yuehong Wei, Yang Wang, Xiaoning Li, Pengzhe Qin, Ying Lu, Jianmin Xu, Shouyi Chen, Meixia Li, Zhicong Yang

**Affiliations:** 1 Guangzhou Center for Disease Control and Prevention, Guangzhou, Guangdong province, China; 2 The First Affiliated Hospital of Guangzhou University of Chinese Medicine, Guangzhou, Guangdong province, China; University of Minnesota, UNITED STATES

## Abstract

**Background:**

The epidemic tendency of hemorrhagic fever with renal syndrome (HFRS) is on the rise in recent years in Guangzhou. This study aimed to explore the associations between meteorological factors and HFRS epidemic risk in Guangzhou for the period from 2006–2015.

**Methods:**

We obtained data of HFRS cases in Guangzhou from the National Notifiable Disease Report System (NNDRS) during the period of 2006–2015. Meteorological data were obtained from the Guangzhou Meteorological Bureau. A negative binomial multivariable regression was used to explore the relationship between meteorological variables and HFRS.

**Results:**

The annual average incidence was 0.92 per 100000, with the annual incidence ranging from 0.64/100000 in 2009 to 1.05/100000 in 2012. The monthly number of HFRS cases decreased by 5.543% (95%CI -5.564% to -5.523%) each time the temperature was increased by 1°C and the number of cases decreased by 0.075% (95%CI -0.076% to -0.074%) each time the aggregate rainfall was increased by 1 mm. We found that average temperature with a one-month lag was significantly associated with HFRS transmission.

**Conclusions:**

Meteorological factors had significant association with occurrence of HFRS in Guangzhou, Southern China. This study provides preliminary information for further studies on epidemiological prediction of HFRS and for developing an early warning system.

## Author summary

The prevalence of HFRS was on the rise in recent years, especially in the large and medium-sized cities in China. We obtained data of HFRS cases in Guangzhou from the National Notifiable Disease Report System (NNDRS) during the period of 2006–2015. Meteorological data were obtained from the Guangzhou Meteorological Bureau. A negative binomial multivariable regression was used to explore the relationship between meteorological variables and HFRS. Meteorological factors had significant association with occurrence of HFRS in Guangzhou, Southern China. This study provides preliminary information for further studies on epidemiological prediction of HFRS and for developing an early warning system.

## Introduction

Hemorrhagic fever with renal syndrome (HFRS) is a rodent-borne zoonosis caused by different species of hantaviruses, characterized by varying degrees of bleeding diathesis, hypertension, and renal failure [1]. In Asia the majority of reported cases of HFRS have occurred in China and the annual incidence of HFRS of China has ranked the highest in the world since 2000 [2]. The prevalence of HFRS peaked in 1986, declined in the 1990s, but it was on the rise in recent years, especially in the large and medium-sized cities [3]. Guangzhou, as a political, economic and cultural center, has over 7.94 million registered inhabitants and 4.76 million floating population (from 2010 census data). Elucidating the dynamic tendencies and influencing factors in Guangzhou will be critical and urgent for developing an appropriate plan for the prevention and control of HFRS.

The epidemiological characteristics of HFRS are affected by various factors, including meteorological factors [4, 5], rodent density [4] and vaccination [6]. Meteorological factors may influence the incidence of HFRS via affecting infection rates and population dynamics of hosts, the regeneration of mites, and the contact rate between rodents and human beings. Infection rates and population dynamics of hosts are thought to be influenced by climatic factors [7–10]. For example, a longitudinal study in Qingdao, China found that precipitation and relative humidity were positively correlated to the densities of hosts and/or hantavirus-positive hosts and the densities of hosts or hantavirus positive hosts were positively correlated to the incidence of HFRS [11]. The data were quite consistent with other reports [12–14].

In China, HFRS is caused mainly by two types of hantavirus, Hantaan virus (HTNV) and Seoul virus (SEOV), previous studies showed that HTNV could be isolated from gamasid and trombiculid mites collected from the nests of field hosts and from laboratory-reared offspring of these mites and that both trombiculid and gamasid mites could transmit HTNV by biting susceptible mammals [15–16].Hantavirus transmission among hosts is speculated to be likely maintained through biting during aggressive interaction [17]. Previous studies indicated that the densities of hosts’ mites could be influenced by humidity, humid environment facilitates the survival or breeding of mites, might be important in mediating host-to-host and possibly host-to-human transmission of hantaviruses [11]. Besides, meteorological factors can influence human behaviors, and thus influence the chance of people having contact with rodent excrement.

Although several studies have explored the associations between meteorological factors and HFRS epidemic risk [18–21], there has been inconsistency between the results due to different models and regions. The most appropriate model for HFRS still remains unclear. One of the critical reasons may be that the variability in meteorological factors in different degrees can produce different effects. The climate of Guangzhou is humid subtropical, where the summer is wet with high temperatures and a high humidity index. Therefore, there is an urgent need to explore the associations between meteorological factors and HFRS epidemic risk in Guangzhou which can help to establish an early warning system for HFRS.

## Methods

### Ethics statement

This study was approved by the Ethics Committee of Guangzhou center for disease control and prevention (GZCDC).

### Setting

**Fig 1:**
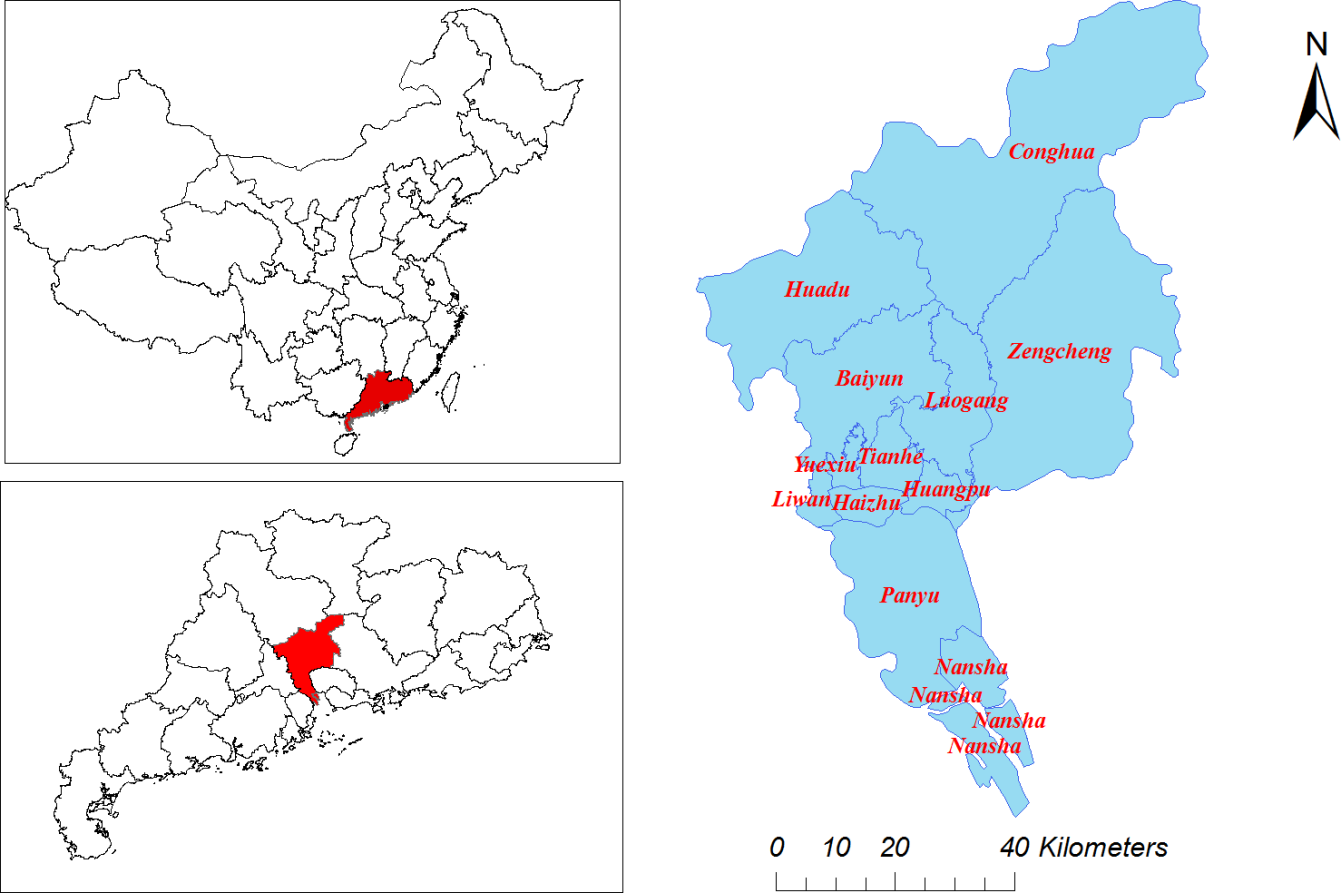
Geographic location of Guangzhou, Guangdong province, China. (Created by ArcGIS 10.1(Environmental Systems Research Institute, Inc)).

Guangzhou (Fig 1), the capital city of Guangdong province of China, is located between longitudes 112°57'E and 114°3'E, latitudes 22°26'N and 23°56'N. The city population in 2015 was 13.50 million. It is situated in the northern hemisphere with an annual average relative humidity of 78%, temperature of 22.3°C and rainfall 2471.9 mm. The climate is humid subtropical, with a wet summer is wet of high temperatures and a high humidity index.

### Surveillance data of HFRS

We obtained data of HFRS cases in Guangzhou from the National Notifiable Disease Report System (NNDRS) during the period of 2006–2015 in this study. All cases of HFRS were diagnosed according to the unified diagnosed criteria issued by Chinese Ministry of Health. The diagnostic principles include epidemiological exposure histories, clinical manifestations and laboratory test. The criteria for probable cases of HFRS include epidemiological exposure histories (traveled to an endemic area or contact with rodents or the urine, droppings, or saliva of infected rodents within 2 months before onset of the disease), clinical manifestations (such as fever, chills, nausea, flushing of the face, inflammation or redness of the eyes, or a rash, low blood pressure and acute kidney failure) and serologic test results positive for hantavirus infection, evidence of hantavirus antigen in tissue by immunohistochemically staining and microscope examination, or evidence of hantavirus RNA sequences in blood or tissue. All the laboratory tests were completed by GZCDC using the same method and same kits. All hospitals and clinics in Guangzhou city are obliged to report HFRS cases through NNDRS within 24 hours.

### Meteorological data

Meteorological data, including daily average temperature (in degrees Centigrade), maximum temperature, minimum temperature, relative humidity (as a percentage), atmospheric pressure (in hPa), wind velocity (in meters per second), sunshine (in hours of daylight) and rainfall (in millimeter) were obtained from the Guangzhou Meteorological Bureau. Monthly meteorological data, including average temperature, cumulative rainfall, average atmospheric pressure, average relative humidity, average wind velocity and cumulative sunshine were calculated.

### Data analysis

The Pearson's correlation coefficients were calculated to examine the degree of multi-collinearity among the meteorological variables. Multi-collinearity was identified when the Pearson's correlation coefficient was greater than 0.9. Given the data were over-dispersed, a negative binomial multivariable regression was used to explore the relationship between meteorological variables and HFRS. The monthly incidence of HFRS was presented as cases per 100000 inhabitants. Meteorological variables for the months preceding the HFRS outbreaks have been shown to be critical. Considering the lagged effect of the meteorological variables on the number of HFRS cases, we incorporated meteorological variables over a range of lags into the regression model. The basic expressions for the model are as follows:

$$log\mu =intercept+b_{1}X_{1}+b_{2}X_{2+}\ldots_{+}b_{m}X_{m},$$

$$\mu =exp\left(intercept+b_{1}X_{1}+b_{2}X_{2+}\ldots_{+}b_{m}X_{m}\right)$$

We calculated the percent increase, which indicated the influences of meteorological variables. All estimates of percent increase were complemented by a 95% confidence interval (CI) and *p*-value. The year variable was forced into the model to eliminate the effects of the long-term trends. All of these analyses were performed using R Project 3.0.2 (R Development Core Team, 2012). The geographic location of Guangzhou was created by ArcGIS 10.1(Environmental Systems Research Institute, Inc).

## Results

### Descriptive analysis

**Fig 2:**
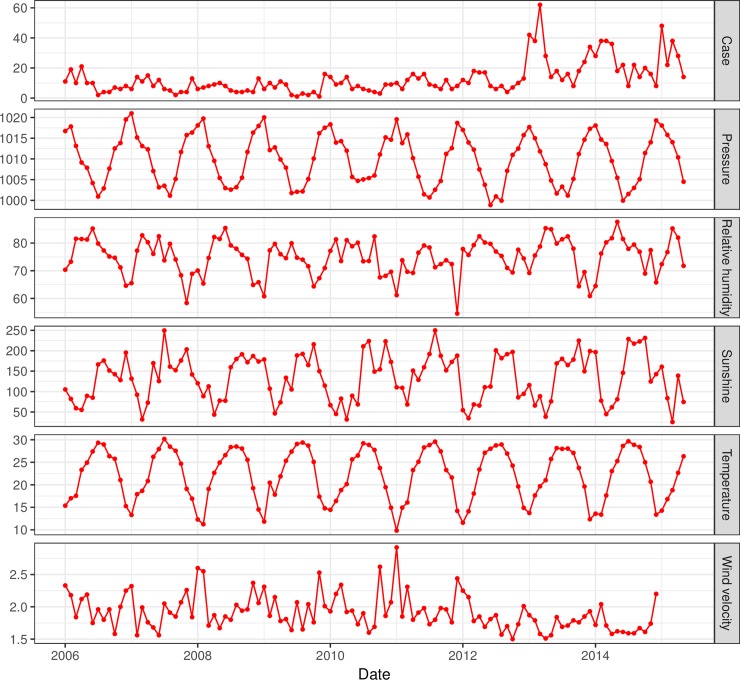
The time series of case and meteorological.

There were 1098 HFRS cases reported in Guangzhou between 2006 and 2015. The annual average incidence was 0.92 per 100000, with the annual incidence ranging from 0.64/100000 in 2009 to 1.05/100000 in 2012. A seasonality phenomenon became apparent, with epidemic peaks occurring in February to May. The peak accounted for 46.45% of all HFRS cases. The monthly average temperature, average atmospheric pressure, average relative humidity, average wind velocity, aggregate rainfall and aggregate sunshine ranged from9.83°C to 30.18°C, from 998.87 hPa to 1020.99 hPa, from 1.77% to 87.65%, from 1.50 m/s to 2.92 m/s, from 0.28 mm to 888.95 mm, respectively (Table 1). The time series of case and meteorological data are shown in Fig 2.

**Table 1:** Summary statistics for monthly confirmed cases and weather conditions in Guangzhou, southern China, 2006–2015.

	Mean	S.D.	Min	P(25)	Median	P(75)	Max
Average temperature (°C)	22.11	5.70	9.83	17.46	23.27	27.48	30.18
Average atmospheric pressure (hPa)	1010.00	5.97	998.87	1004.75	1011.07	1014.84	1020.99
Average relative humidity (%)	73.61	11.62	1.77	69.85	75.71	79.77	87.65
Average wind velocity (m/s)	1.91	0.27	1.50	1.71	1.85	2.05	2.92
Aggregate rainfall (mm)	162.55	159.79	0.28	43.76	135.00	224.77	888.95
Aggregate sunshine (h)	133.51	57.35	25.22	82.23	141.83	179.20	249.82
Confirmed cases	9.35	5.41	1.00	5.00	8.00	12.00	30.00

Table footnotes: all the data were presented as monthly average or aggregate values.

S.D. = Standard deviation.

### Correlation analysis

The Pearson's correlation coefficients revealed a strong correlation (r = -0.937, *P*<0.01) between average temperature and average atmospheric pressure (Table 2). The result indicated that collinearity of the preliminary variables can be observed in our study.

**Table 2:** Pearson’s correlation coefficient(r) matrix of meteorological variables and cases in Guangzhou, southern China, 2006–2015.

	Atmospheric pressure	Relative humidity	Average temperature	Rainfall	Sunshine	Wind velocity
Atmospheric pressure	1					
Relative humidity	-0.270**	1				
Average temperature	-0.937**	0.206*	1			
Rainfall	-0.628**	0.160	0.521**	1		
Sunshine	-0.255**	-0.224*	0.394**	-0.220*	1	
Wind velocity	0.536**	-0.529**	-0.547**	-0.342**	-0.089	1
Case	0.248**	-0.007	-0.314**	-0.066	-0.298**	-0.270**
One-month lag cases	0.248**	-0.007	-0.314**	-0.066	-0.298**	-0.270**
Two-month lag cases	0.401**	-0.064	-0.449**	-0.264**	-0.222*	-0.025
Three-month lag cases	0.397**	-0.154	-0.429**	-0.314**	-0.082	-0.092
Four-month lag cases	0.332**	-0.275**	-0.326**	-0.272**	0.116	-0.065

*P<0.05

** P<0.01.

### Negative binomial regression

**Fig 3:**
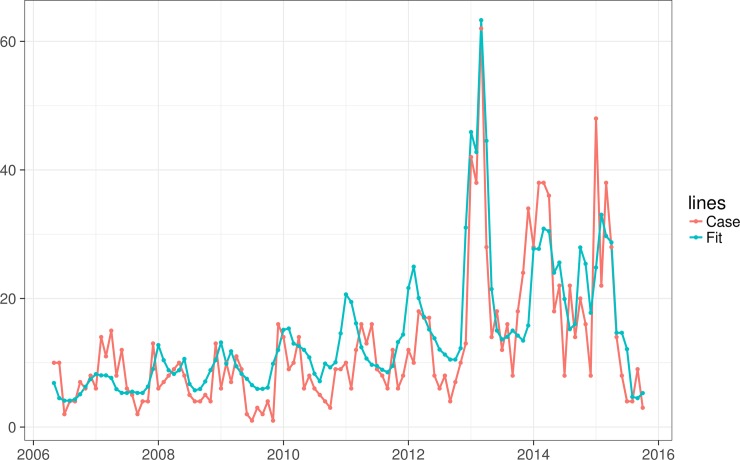
The compare of fitted and cases in the final model.

In this study, lags of meteorological variables from one to four months were included to build different models, and to avoid the collinearity of average temperature and average atmospheric pressure, we put them into two different models when exploring the relationship between meteorological variables and HFRS. There were significant lag effects between meteorological variables and monthly cases of HFRS (Table 3). Average temperature and aggregate rainfall in the same month, lags of average temperature from one to three months, aggregate rainfall of two months and average relative humidity of four months all have significant association with the incidence of HFRS. The final negative binomial regression model (Table 4) suggests that the monthly number of HFRS cases decreased by 5.543% (95%CI -5.564% to -5.523%) each time the temperature was increased by 1°C, and the number of cases decreased by 0.075% (95%CI -0.076% to -0.074%) each time the aggregate rainfall was increased by 1 mm. The comparison of fitted and cases in the final model is shown in Fig 3.

**Table 3:** Negative binomial regression model of meteorological factors associated with risk of HFRS incidence in Guangzhou, southern China, 2006–2015.

	Lag0	Lag1	Lag2	Lag3	Lag4
Average relative humidity	0.795	1.020	1.119	-1.874	-2.381*
	(0.78~0.804)	(1~1.031)	(1.097~1.138)	(-1.837~-1.853)	(-2.405~-2.358)
Aggregate rainfall	-0.075 *	-0.03	-0.120*	-0.053	-0.056
	(-0.076~-0.074)	(-0.037~-0.037)	(-0.119~-0.118)	(-0.052~-0.052)	(-0.057~-0.055)
Average temperature	-5.543**	-5.995 **	-4.568 **	-2.564 *	0.840
	(-5.432~-5.523)	(-5.874~-5.976)	(-4.477~-4.549)	(-2.513~-2.543)	(0.818~0.862)
Year	16.883*	16.381 *	16.661 *	17.810 *	19.006*
	(16.577~16.919)	(16.084~16.415)	(16.359~16.696)	(17.488~17.848)	(18.964~19.047)

Table footnotes: Percent increase = (e^β^-1)*100, 95%CI for percent increase (%), CI = Confidence interval

*P<0.05

** P<0.01.

**Table 4:** Parameters estimated by final negative binomial regression model for HFRS in Guangzhou, southern China, 2006–2015.

Variable	Percent increase	95%CI	P-value
Average relative humidity,4-month lag	0.795	0.787–0.804	0.061
Aggregate rainfall, 0-month lag	-0.075	-0.076–0.074	0.039
Average temperature,1-month lag	-5.543	-5.564–5.523	<0.01
Year	16.883	16.846–16.919	<0.01

## Discussion

HFRS is epidemic in many provinces in mainland China, and it is worth noting that the prevalence is on the rise in the large and medium-sized cities, such as Guangzhou. Transmission of hantaviruses from rodents to humans is believed to occur through inhalation of aerosols contaminated by virus shed in excreta, saliva, and urine of infected animals [22–24]. The number of HFRS cases is influenced by the density and hantavirus infection rate of host rodents, as well as the contact rate between rodents and human beings [7, 25]. Meteorological factors may influence the incidence of HFRS via affecting infection rates and population dynamics of hosts, the regeneration of mites, and the contact rate between rodents and human beings. Vector-borne viral diseases including HFRS are amongst the most sensitive of all diseases to climate change [26]. Climate change would directly affect disease transmission by shifting the reservoir's geographical range and increasing reproductive rates and by shortening the pathogen's incubation period [10].

The results of this study show that average temperature was negatively associated with the incidence of HFRS, which is in agreement with the results at Shandong [27]. However, inconsistent findings have also been reported in other studies. The results of a study in Junan County showed that extremes of weather (too cold or too hot) do not favor HFRS prevalence, and the most appropriate mean temperature was between 10°C and 25°C [13] Lin et al. applied a generalized additive model to examine the effect of meteorological factors on the occurrence of HFRS in Jiaonan county, China [28]. They found that a daily mean temperature at about 17°C was associated with highest HFRS occurrence, a positive association between temperature and HFRS occurrence was observed when the daily mean temperature was below 17°C, while when the daily mean temperature was higher than 17°C, an inverse association was observed. Temperature could affect the breeding and survival of rodents as well as infectivity of hantavirus; it could also affect the activities of both rodents and the human population. In cooler climates, warmer temperatures may allow reservoirs to survive more easily in winters that normally would have limited their populations and to cause rodents to reach maturity much faster than lower temperatures [21]. The discrepancy might be due to the difference in the characteristics of climate of the study regions and different models applied in the studies. In Guangzhou, the mean temperature is 22.11°C, which is close to the highest suitable temperature for outside activities of both rats and human (25°C), so in this area, when the temperature increases further, there is less interaction between rodents and humans, leading to the decrease of the disease.

The results of this study show that there is a 1 month lagged effect of average temperature to the incidence of HFRS. The lag would capture the period of rodents growth, virus development time within the rodents and the virus incubation period within the human body [10]. This lead time is of practical importance in predicting epidemics of HFRS and giving health authorities sufficient time to formulate plans, disseminate warnings, and implement public health interventions, such as vaccinating high-risk populations, killing the rodent hosts, and managing environments for the prevention and control of the disease (Ministry of Health 1998) [21]. While the lag effects for associations of the climatic variables were inconsistent, for example the study of Elunchun and Molidawahaner showed that land surface temperature, rainfall and relative humidity were significantly correlated with the monthly reports of HFRS with lags of 3–5 months [21], and the study in Heilongjiang Province showed an important seasonal signal in monthly maximum temperature, relative humidity with a lag of 1–3 months in the association with reported HFRS cases [18]. The difference may due to geographical difference, where the meteorological characteristics and biological characteristics of viral transmission may be different.

The results of this study suggest that aggregate rainfall have significant association with the incidence of HFRS, which is consistent with the findings of previous studies. Fang et al. found that there was a negative association between monthly cumulative precipitation and HFRS, in a study carried out in Shandong Province [27], and similar results were also reported in Yingshang County [10], Jiangsu Province and Jiaonan County [28], China. Although heavy precipitation followed by increased grass seed production was associated with higher deer mouse densities that caused an outbreak of hantavirus pulmonary syndrome in the Four Corners region of the USA [29–32], excessive rainfall could have a negative impact on rodents by destroying their habitats [10,33]. In addition, frequent rain may decrease the likelihood of rodent-to-rodent contact, rodent-to-human contact, and virus transmission due to decreased rodent activity and reduced human exposure [10].

The present study is first to investigate the effect of meteorological factors on HFRS incidence in southern China, to the best of our knowledge. However, some limitations should be noted when interpreting findings from this study. Firstly, the study design was an ecological study; it did not allow us to explore individual-based association and limited the capacity for causal inference. Secondly, the occurrence of HFRS cases in each region may not be caused by climate alone. Other factors such as human activities and movement, socioeconomics status, land use and population immunity may contribute to the transmission of HFRS. However, data are limited on many of these variables. Thus we could not exclude these potential confounding factors. Therefore, further studies of the associations between meteorological factors and occurrence of HFRS are warranted.

In conclusion, this study demonstrated that meteorological factors had significant association with occurrence of HFRS in Guangzhou, Southern China. A rise in temperature and rainfall may reduce the risk of HFRS infection. This study provides preliminary information for further studies on epidemiological prediction of HFRS and for developing an early warning system.

## Supporting information

S1 File File on HFRS confirmed cases in Guangzhou, southern China, 2006–2015. (XLSX)

## References

[1] Sargianou M, Watson DC, Chra P, Papa A, Starakis I, Gogos C, et al Hantavirus infections for the clinician: from case presentation to diagnosis and treatment. Critical Reviews in Microbiology. 2012;38(4):317–29. 10.3109/1040841X.2012.673553 22553984

[2] Watson DC, Sargianou M, Papa A, Chra P, Starakis I, Panos G. Epidemiology of Hantavirus infections in humans: A comprehensive, global overview. Critical Reviews in Microbiology. 2014;7828(3):261–72. 10.3109/1040841X.2013.783555 23607444

[3] Zhang YZ, Xiao DL, Wang Y, Wang HX, Sun L, Tao XX, et al [The epidemic characteristics and preventive measures of hemorrhagic fever with syndromes in China]. Zhonghua liu xing bing xue za zhi = Zhonghua liuxingbingxue zazhi. 2004;25(6):466 15231118

[4] Xiao H, Gao LD, Li XJ, Lin XL, Dai XY, Zhu PJ, et al Environmental variability and the transmission of haemorrhagic fever with renal syndrome in Changsha, People's Republic of China. Epidemiology & Infection. 2013;141(9):1867 10.1017/S0950268812002555 23158456 PMC9151413

[5] Viel JF, Lefebvre A, Marianneau P, Joly D, Giraudoux P, Upegui E, et al Environmental risk factors for haemorrhagic fever with renal syndrome in a French new epidemic area. Epidemiology & Infection. 2011;139(6):867–74. 10.1017/S0950268810002062 20822577

[6] Xiao D, Wu K, Tan X, Yan T, Li H, Yan Y. The impact of the vaccination program for hemorrhagic fever with renal syndrome in Hu County, China. Vaccine. 2014;32(6):740–5. 10.1016/j.vaccine.2013.11.024 24252696

[7] Zhang WY, Fang LQ, Jiang JF, Hui FM, Glass GE, Yan L, et al 2009 Predicting the risk of hantavirus infection in Beijing, People’s Republic of China. Am J Trop Med Hyg 80(4):678–683. 19346399

[8] Glass GE, Shields T, Cai B, Yates TL, Parmenter R. 2007 Persistently highest risk areas for hantavirus pulmonary syndrome: potential sites for refugia. Ecol Appl 17(1):129–139. 17479840 10.1890/1051-0761(2007)017[0129:phrafh]2.0.co;2

[9] Yan L, Fang LQ, Huang HG, Zhang LQ, Feng D, Zhao WJ, et al 2007 Landscape elements and Hantaan virus-related hemorrhagic fever with renal syndrome, People’s Republic of China. Emerg Infect Dis 13(9):1301–1306. 10.3201/eid1309.061481 18252099 PMC2857277

[10] Bi P, Tong S, Donald K, Parton K, Ni J. Climatic, reservoir and occupational variables and the transmission of haemorrhagic fever with renal syndrome in China. International Journal of Epidemiology. 2002;31(1):189 11914320 10.1093/ije/31.1.189

[11] Jiang F, Wang L, Wang S, et al Meteorological factors affect the epidemiology of hemorrhagic fever with renal syndrome via altering the breeding and hantavirus-carrying states of rodents and mites: a 9 years’ longitudinal study. *Emerging Microbes & Infections*. 2017;6(11):e104 10.1038/emi.2017.92. 29184158 PMC5717093

[12] Jiang F, Zhang Z, Dong L et al Prevalence of hemorrhagic fever with renal syndrome in Qingdao City, China, 2010–2014. Sci Rep 2016; 6: 36081 10.1038/srep36081. 27786303 PMC5081555

[13] Liu J, Xue FZ, Wang JZ, Liu QY. Association of haemorrhagic fever with renal syndrome and weather factors in Junan County, China: a case-crossover study. Epidemiol Infect 2013; 141: 697–705. 10.1017/S0950268812001434. 22793368 PMC9151836

[14] Tian HY, Yu PB, Luis AD et al Changes in rodent abundance and weather conditions potentially drive hemorrhagic fever with renal syndrome outbreaks in Xi'an, China, 2005–2012. PLoS Negl Trop Dis 2015; 9: e0003530 10.1371/journal.pntd.0003530. 25822936 PMC4378853

[15] Yu XJ, Tesh RB. The role of mites in the transmission and maintenance of Hantaan virus (Hantavirus: Bunyaviridae). J Infect Dis 2014; 210: 1693–1699. 10.1093/infdis/jiu336 24958909 PMC4296190

[16] Wu G, Zhang Y, Guo H, Jiang K, Zhang J, Gan Y. The role of Leptotrombidium scutellare in the transmission of human diseases. Chin Med J (Engl) 1996; 109: 670–673. 9275333

[17] Glass GE, Childs JE, Korch GW, LeDuc JW. Association of intraspecific wounding with hantaviral infection in wild rats (Rattus norvegicus). Epidemiol Infect 1988; 101: 459–472. 3141203 10.1017/s0950268800054418 PMC2249393

[18] Li CP, Cui Z, Li SL, Magalhaes RJ, Wang BL, Zhang C, et al Association between hemorrhagic fever with renal syndrome epidemic and climate factors in Heilongjiang Province, China. American Journal of Tropical Medicine & Hygiene. 2013;89(5):1006 10.4269/ajtmh.12-0473 24019443 PMC3820312

[19] Xiao H, Tian HY, Cazelles B, Li XJ, Tong SL, Gao LD, et al Atmospheric Moisture Variability and Transmission of Hemorrhagic Fever with Renal Syndrome in Changsha City, Mainland China, 1991–2010. Plos Negl Trop Dis. 2013;7(6):e2260 10.1371/journal.pntd.0002260 23755316 PMC3674989

[20] Han SS. Air pollution and hemorrhagic fever with renal syndrome in South Korea: an ecological correlation study. BMC Public Health. 2013;13(1):1–6. 10.1186/1471-2458-13-347 23587219 PMC3641006

[21] Zhang WY, Guo WD, Fang LQ, Li CP, Bi P, Glass GE, et al Climate Variability and Hemorrhagic Fever with Renal Syndrome Transmission in Northeastern China. Environmental Health Perspectives. 2010;118(7):915–20. 10.1289/ehp.0901504 20142167 PMC2920909

[22] Clement JP. 2003 Hantavirus. Antiviral Res 57(1–2):121–127. 12615308 10.1016/s0166-3542(02)00205-x

[23] Shi LY. 2003 Epidemiology [in Chinese]. Beijing: China People’s Health Publishing House.

[24] Song G. 1999 Epidemiological progresses of hemorrhagic fever with renal syndrome in China. Chin Med J (Engl) 112(5):472–477. 11593522

[25] Jiang JF, Wu XM, Zuo SQ, Wang RM, Chen LQ, Wang BC, et al 2006 Study on the association between hantavirus infection and *Rattus norvegicus* [in Chinese]. Chin J Epidemiol 27(3):196–199. 16792882

[26] Mackenzie J. Vector-borne viral diseases and climate change in the Australasian region: Major concerns and the public health response. 1997.

[27] Fang LQ, Wang XJ, Liang S, Li YL, Song SX, Zhang WY, et al Spatiotemporal Trends and Climatic Factors of Hemorrhagic Fever with Renal Syndrome Epidemic in Shandong Province, China. Plos Neglected Tropical Diseases. 2010;4(8):e789 10.1371/journal.pntd.0000789 20706629 PMC2919379

[28] Lin H, Zhang Z, Lu L, Li X, Liu Q. Meteorological factors are associated with hemorrhagic fever with renal syndrome in Jiaonan County, China, 2006–2011. International Journal of Biometeorology. 2014;58(6):1031–7. 10.1007/s00484-013-0688-1 23793957

[29] Engelthaler DM, Mosley DG, Cheek JE, Levy CE, Komatsu KK, et al (1999) Climatic and environmental patterns associated with hantavirus pulmonary syndrome, Four Corners region, United States. Emerg Infect Dis 5: 87–94. 10.3201/eid0501.990110 10081675 PMC2627709

[30] Hjelle B, Glass GE (2000) Outbreak of hantavirus infection in the Four Corners region of the United States in the wake of the 1997–1998 El Nino-southern oscillation. J Infect Dis 181: 1569–1573. 10.1086/315467 10823755

[31] Tamerius J, Wise E, Uejio C, McCoy A, Comrie A (2007) Climate and human health: synthesizing environmental complexity and uncertainty. Stoch Environ Res Risk Assess 21: 601–613. 10.1007/s00477-007-0142-1

[32] Semenza JC, Menne B (2009) Climate change and infectious diseases in Europe. Lancet ID 9: 365–375. 10.1016/S1473-3099(09)70104-5 19467476

[33] Bi P (2003) El Nin˜o and incidence of hemorrhagic fever with renal syndrome in China, JAMA 289: 176–177. 12517227 10.1001/jama.289.2.176-d

